# Chancery Lunatics

**Published:** 1851-10-01

**Authors:** 


### CHANCERY LUNATICS.

We have before us a pamphlet, entitled "*A Letter addressed to the Committee of the House of Lords, sitting on the Master's Jurisdiction, &c.*," signed, "A Suitor in Chancery," dated "Bath, July 28, 1851." As this document only reached us just as we were going to press, we are prevented from making any comments either upon the facts it contains, or the deductions and suggestions of the writer. We can only, in this number of our Journal, make one or two extracts from the pamphlet. In referring to appointments of "*committees to the person*" of Chancery lunatics, the author observes:

"The extent of this patronage may be surmised by a reference to the Parliamentary Paper, No. 505 of this year.

"In 1843 the Masters had to appoint 28 Committees.

| | | | | |
|---|---|---|---|---|
| 44 | ,, | ,, | 30 | ,, |
| 45 | ,, | ,, | 20 | ,, |
| 46 | ,, | ,, | 23 | ,, |
| 47 | ,, | ,, | 31 | ,, |
| 48 | ,, | ,, | 18 | ,, |
| 49 | ,, | ,, | 30 | ,, |
| | | | 180 | |

"All the cognisance taken by the Court of Chancery of the money entrusted to Committees will be understood by the following extracts from the annual accounts of the Committee of the Estate of two different lunatics.

"'Retained for the Committee of the person, on account of twelve months' maintenance, at the rate of £374 per annum:

| | |
|---|---|
| "'From the 1st of February, 1850, to January 30, 1851 . | £374 0 0 |
| "'Pocket money, at the rate of £20 per annum . . . . | 20 0 0 |
| "'For clothes, at the rate of £30 per annum . . . . . | 30 0 0 |
| | £424 0 0' |

"But not a single voucher of this expenditure is produced, although applied for when appearing before the Master, and whether the whole or half the money is laid out, is not inquired into. Again, in another case:

'Retained on account of two years' maintenance, from November, 1848, to November, 1850, at the allowance of £400 per annum . . . £800 0 0'

"The same Parliamentary paper shows that to these persons an income of £65,000 was intrusted in various amounts for the care of the 180 persons made lunatic wards of Chancery during that period."

When speaking of the power possessed by the "Committee" over the *locus in quo* of the Chancery lunatic, our author remarks:—

"These individuals possess an authority greater than that of a father over an infant, for, as I already stated, they render no pecuniary account of what they disburse to any earthly being, not even to the court which appointed them, while the extent of liberty or restraint is solely with them—they can send the invalid to any asylum they please, without consulting the heir at law, or the Lord Chancellor's visitors in lunacy; they can keep him in the house of a medical man objected to by the invalid himself, as well as by his nearest relatives,—they go further,—they clothe him as they please—they dole out, according to their liberality, the few shillings he may solicit permission to disburse, or be allowed out of his fortune, and as they have the selecting of the persons by whom the invalid is surrounded, they control a mass of evidence which enables them, under the plea of its producing excitement, to discard relatives from the house of the invalid, to prevent him seeing his friends, or to have any communication, even by letter, with any of them; they refuse him permission even to go to the house of a brother, when that brother is willing to receive him, nor has the master any power to grant the wishes of the lunatic against the fiat of the committee. It can only be obtained by petition to the Chancellor."

Can the following statement be verified? If so, the matter requires looking into.

"Few of the committees disburse *more than two-thirds* of the allowance for maintenance on the invalid intrusted to their care, many of them less, and none ever restore these victims to the society from which they were intended only to be temporarily removed. Of the 537 Chancery lunatics, 238 are placed by the committees of the person in lunatic asylums, others in private residences. A few only are living with their relatives or friends."

The following returns will give an insight of the number of Chancery lunatics—the vast and annual amount of their real property—the allowances granted to committees for their maintenance—and the number of those who have been made lunatic wards of Chancery, but whose fortunes were not ascertained, and the allowance for maintenance fixed.

| Years. | Number of Wards of Chancery, whose Incomes are known. | Number of Arrears of those whose allowances were not fixed by the Court. |
|---|---|---|
| 1832 | 386 | 43 |
| 1833 | 399 | 48 |
| 1839 | 494 | 61 |
| 1849 | 531 | 39 |

Return, dated 5th June, 1832 (the first of the Returns to Parliament), of the number of Lunatics confined under the authority of the Lord Chancellor.

| | | |
|---|---|---|
| 109 | lunatics whose property amounts to less than £200 per annum each: Total annual amounts of such property | £11,210 14 3 |
| 234 | lunatics whose property amounts to £200 each per annum and upwards: Total annual amounts of such property | 264,464 14 7 |
| 43 | lunatics whose income is not ascertained | £275,675 8 10 |
| 386 | | |

A Return, (dated 7th March, 1833,) made up to the latest possible period, of the Number of Lunatics confined under the authority of the Crown, and of the Total Amount of their Annual Incomes.

| | | |
|---|---|---|
| | There are 399 lunatics, confined under the authority of the Crown, the total of whose annual incomes amounts to | £269,158 1 9 |
| | Of which number there are: | |
| 57 | who individually have less than £100 per annum, and whose incomes amount to | 3,254 11 9 |
| 61 | who have £100, and less than £200 per annum, and whose incomes amount to | 8,675 2 0 |
| 50 | who have £200, and less than £200 per annum, and whose incomes amount to | 12,130 0 3 |
| 31 | who have £300, and less than £400 per annum, and whose incomes amount to | 10,050 15 10 |
| 152 | who have £400 per annum and upwards, and whose incomes amount to | 235,047 11 11 |
| | and | |
| 48 | whose incomes are not ascertained. | £269,158 1 9 |
| 9 | | |

Return to an Order of the Honourable House of Commons, (dated 21st Feb., 1839.) Returns made up to the latest possible period of the number of Lunatics against whom Commissions of Lunacy are now in force, and of the Total Amount of their Annual Incomes, and the Total Amount of the Sums allowed for their Maintenance.

| | | Income. | Maintenance. |
|---|---|---|---|
| 86 | Persons who individually have less than £100 per annum, and whose incomes amount to | £4,654 15 10 | |
| | And the total of the sums allowed for their maintenance to | | £4,632 0 4 |
| 83 | Who individually have more than £100, and less than £200 per annum, and whose incomes amount to | 11,702 16 9 | |
| | And the total of the sums allowed for their maintenance to | | 8,960 5 4 |

| | | Income. | Maintenance. |
|---|---|---|---|
| 98 | Who individually have more than £200, and less than £400 per annum, and whose incomes amount to . . . . . . . . . . | £26,567 6 1 | |
| | And the total of the sums allowed for their maintenance to . . . . . . . . . . . . . . . . . | | 20,728 4 8 |
| 49 | Persons who individually have more than £400, and less than £600 per annum, and whose incomes amount to . . . . . . | 24,700 9 1 | |
| | And the total of the sums allowed for their maintenance to . . . . . . . . . . . . . . . . . | | 17,451 8 7 |
| 46 | Persons who individually have more than £600, and less than £1000 per annum, and whose incomes amount to . . . . | 36,195 13 7 | |
| | And the total of the sums allowed for their maintenance to . . . . . . . . . . . . . . . . . | | 25,764 7 0 |
| 71 | Persons who individually have more than £1000 per annum, and whose incomes amount to . . . . . . . . . . . | 174,170 11 11 | |
| | And the total of the sums allowed for their maintenance to . . . . . . . . . . . . . . . . . | | 91,551 14 9 |
| 61 | Of the 494 persons above mentioned, many are recent cases, and the number where the fortune is not yet ascertained, and the allowance for maintenance fixed, is 61 | | |
| 494 | | £277,991 13 3 | £169,388 0 8 |

20 *June*, 1839.

---

Return to an Order of the Honourable the House of Commons, (dated 28th August, 1848.) Returns, made up to the latest possible period, of the Number of Lunatics against whom Commissions of Lunacy are now in force, and of the Total Amount of their Annual Incomes, and the Total Amount of the Sums allowed for their Maintenance, (in continuation of Parliamentary Paper, No. 78, of Session 1839).

| | | Income. | Maintenance. |
|---|---|---|---|
| | There are 531 persons against whom commissions of lunacy are now in force, the total of whose annual incomes amounts to . . . . . . . . . . . . . . . . | £333,781 8 11 | |
| | And the total of the sums allowed for their maintenance to . . . . . . . . . . . . . . . . . | | £213,074 13 2 |
| 94 | Of the above there are 94 who individually have less than £100 per annum, and whose incomes amount to . . . . . | 5,594 6 6 | |
| | And total amount of the sums allowed for their maintenance to . . . . . . . . . . . . . . . | | 5,228 3 7 |
| 106 | Who individually have more than £100, and less than £200 per annum, and whose incomes amount to . . . . . . . . . . | 15,176 11 10 | |
| | And the total of the sums allowed for their maintenance to . . . . . . . . . . . . . . . . . | | 13,099 4 4 |
| 104 | Who individually have more than £200 and less than £400 per annum, and whose incomes amount to . . . . . . . . . . | 30,214 7 11 | |
| | And the total of the sums allowed for their maintenance to . . . . . . . . . . . . . . . . . | | 23,590 19 10 |
| 63 | Who individually have more than £400 and less than £600 per annum, and whose incomes amount to . . . . . . . . . | 30,033 18 11 | |
| | And the total of the sums allowed for their maintenance to . . . . . . . . . . . . . . . . . | | 23,452 17 0 |

| | | Income. | Maintenance. |
|---|---|---|---|
| 51 | Who individually have more than £600 and less than £1000 per annum, and whose incomes amount to . . . . . . . . . | £39,125 3 0 | |
| | And the total of the sums allowed for their maintenance to . . . . . . . . . . . . . . . . . | | £26,672 3 0 |
| 74 | Who individually have more than £1000 and whose incomes amount to . . . . . | 213,637 0 9 | |
| | And the total of the sums allowed for their maintenance to . . . . . . . . . . . . . . . . . | | 121,121 5 5 |
| 39 | Whose incomes have not yet been ascertained, nor their maintenance fixed. | | |
| 531 | | £333,781 8 11 | £213,074 13 2 |

N.B.—The above Return comprises *all existing Lunatics* by *Inquisition*, without reference to any former Paliamentary Paper.

(Signed) THOMAS CARTLEDGE,

*February 27th*, 1849. Secretary of Lunatics to the Lord Chancellor.

Lord Brougham in 1833 introduced a Bill to diminish the expense of Commissions in the nature of Writs *De Lunatico Inquirendo* and to provide for the better care of Lunatics. Under it, authority is given to the Lord Chancellor to appoint, as visitors of lunatics, three persons, two of whom shall be physicians, and one a barrister of not less than five years' standing. Also, a secretary, with salaries and expenses as follows:—

| | |
|---|---|
| Two physicians £500 a year each . . | £1000 0 0 |
| One barrister, at . . . . . . . . . . | 300 0 0 |
| One secretary . . . . . . . . . . . | 300 0 0 |
| For an office and general expenses . . | 300 0 0 |
| | £1900 0 0 |

Exclusive of travelling expenses, which might be allowed by the Lord Chancellor.

By a Return ordered July 8, 1851, it appears there was paid to this board

| | |
|---|---|
| From June 1844 to January 1845 . . | £3268 14 0 |
| „ 1845 „ 1846 . . | 3043 8 0 |
| „ 1846 „ 1847 . . | 3009 16 0 |
| „ 1847 „ 1848 . . | 2839 10 0 |
| „ 1848 „ 1849 . . | 2756 0 0 |

The Act further says, that each of such persons so found lunatic shall be visited at least once a year, by one of such medical visitors, who, after such visitation, shall respectively make a report to the Lord Chancellor, in writing, of the state of mind, and bodily health and general condition, and of the care and treatment pursued to each such person visited, which reports are to be duly filed and kept secret in the office of such visitors, and shall be open to the inspection of *no person whatever, except* the said visitors, their secretary and the Lord Chancellor, or such as the Lord Chancellor shall appoint.

This Bill was passed in 1833 when the number of Chancery lunatics was 386, their numbers now are about 550.

The board consists of Dr. Southey, Dr. Bright, Mr. Phillimore, barrister, and Mr. Enfield their secretary, who, when the Masters in Lunacy were appointed, became also chief clerk to them, with a salary of £800 a year, in addition to the £300 he previously had.

The pamphlet concludes with some general observations (from some of which we dissent), to which we hope to direct attention in an early number.

